# Points and the Delivery of Gameful Experiences in a Gamified Environment: Framework Development and Case Analysis

**DOI:** 10.2196/35907

**Published:** 2022-09-29

**Authors:** Sungjin Park, Sangkyun Kim

**Affiliations:** 1 Graduate School of Business Kyung Hee University Seoul Republic of Korea

**Keywords:** point, design framework, gamification, gameful experience, pointsification, overjustification effect

## Abstract

**Background:**

Points represent one of the most widely used game mechanics in gamification. They have been used as a means to provide feedback to users. They visually show user performance and are used along with other game mechanics to produce synergy effects. However, using points without analyzing the application environment and targets adversely affects users.

**Objective:**

This study aims to identify the problems that users encounter when points are applied improperly, to solve problems based on an analysis of previous studies and actual point use cases, and to develop a point design framework to deliver gameful experiences.

**Methods:**

Three problems were identified by analyzing previous studies. The first problem is points that only accumulate. The second is points that emphasize a user’s difference from other people. The third pertains to the reward distribution problem that occurs when points are used as rewards.

**Results:**

We developed a framework by deriving 3 criteria for applying points. The first criterion is based on the passive acquisition approach and the active use approach. The second criterion is used to classify points as “high/low” and “many/few” types. The third criterion is the classification of personal reward points and group reward points based on segmentation of the reward criteria. We developed 8 types of points based on the derived point design framework.

**Conclusions:**

We expect that some of the problems that users experience when using points can be solved. Furthermore, we expect that some of the problems that arise when points are used as rewards, such as pointsification and the overjustification effect, can be solved. By solving such problems, we suggest a direction that enables a gameful experience for point users and improves the core value delivery through gameful experiences. We also suggest a gameful experience delivery method in the context of the ongoing COVID-19 pandemic.

## Introduction

### Background

Gamification is a technique that encourages participation and immersion by providing a gameful experience to users in nongame contexts, such as business management, marketing, health care, and education [1]. A gameful experience succeeds when users experience the feeling of playing a game [2] in what is normally a nongame context for them [1]. This is differentiated from a gaming experience, which refers to what an individual feels while playing an actual game; a gameful experience is a game-like experience in a nongame context [2]. A gameful experience enables users’ enjoyment, absorption, creative thinking, absence of negative affect, activation, and dominance [2]. It is an important topic for millennials and Generation Z (which we refer to collectively as “Generation MZ”), who differ from previous generations. They have benefited from technology, have been using smartphones from childhood, and have naturally accessed a variety of content through such devices. Games are a familiar type of content and are often accessed; consequently, people in these generations prefer gameful experiences [3,4].

Generation MZ has been forecast to become the largest consumer base from 2020 to 2035. Kristofer et al [5] analyzed the world’s spending power and predicted the spending power of each generation from 2020 to 2035. According to this work, baby boomers and Generation X will show downward trends from 2020 to 2035, but Generation MZ is expected to continue to show an increasing trend [5].

Hence, many attempts have been made globally to serve Generation MZ, and gamification is one of those attempts. According to Park and Kim [6], education and training account for the largest portion of a total of 754 attempts to apply gamification worldwide, followed by human resource management, social issues, commercialization, and lifestyle.

As gamification began to draw attention, research began on systematizing development methods in order to deliver core values to users with gameful experiences. Gamification development consists of procedures that analyze constituent elements of a game, such as game mechanics [7], game rules, and sensory elements, that connect the player and the game physically and psychologically.

The oldest gamification development method is the point, badge, and leaderboard (PBL) system, which refers to game mechanics. The PBL system is the most widely used and easily applied method in gamification [8]. Park and Kim [6] showed that points are the most widely used game mechanic, and they are most commonly applied in gamification for health care [9] and in the field of education [10].

More factors should be considered than might be expected when applying gamification to deliver gameful experiences to users, including factors affecting the use of points, among other gamification elements. Environmental factors in applying points, user characteristics, and the significance and value of the points used as rewards should be considered. Before applying points, instructors should check if points are usable in a particular class. Additionally, it is necessary to consider whether missions and quests, which are required to give learners points, can be provided. Points are a means to stimulate intrinsic motivation and display the performance of a learner [11]. However, when points are applied without an appropriate purpose, they adversely affect users. Typical problems include “pointsification” and the “overjustification” effect. Pointsification is a phenomenon wherein the user does not perform the activity intended by the developer and acquires points without purpose, thus nullifying the purpose of applying gamification [11]. The overjustification effect is associated with motivation: when users act by their own will, it is because of inner motivation. In contrast, when users do not act on their own, reinforcement is used to increase the probability of the user performing the intended action [12]. By using points as reinforcement, users’ external motivation is stimulated, and internalization of the external motivation is induced through continuous stimulation. The overjustification effect occurs when the internalization of external motivation is absent, and the internal motivation decreases because the user performs actions based only on the reinforcement [13,14].

This study analyzes problems with points reported in previous studies and develops a point-design framework to supplement the points system. Unlike the traditional methodology, the framework can be easily used by users, and it reduces the gap between the field and academic perspectives [15].

### Literature Review

#### History and Effects of Points in Learning Environments

**Figure 1:**
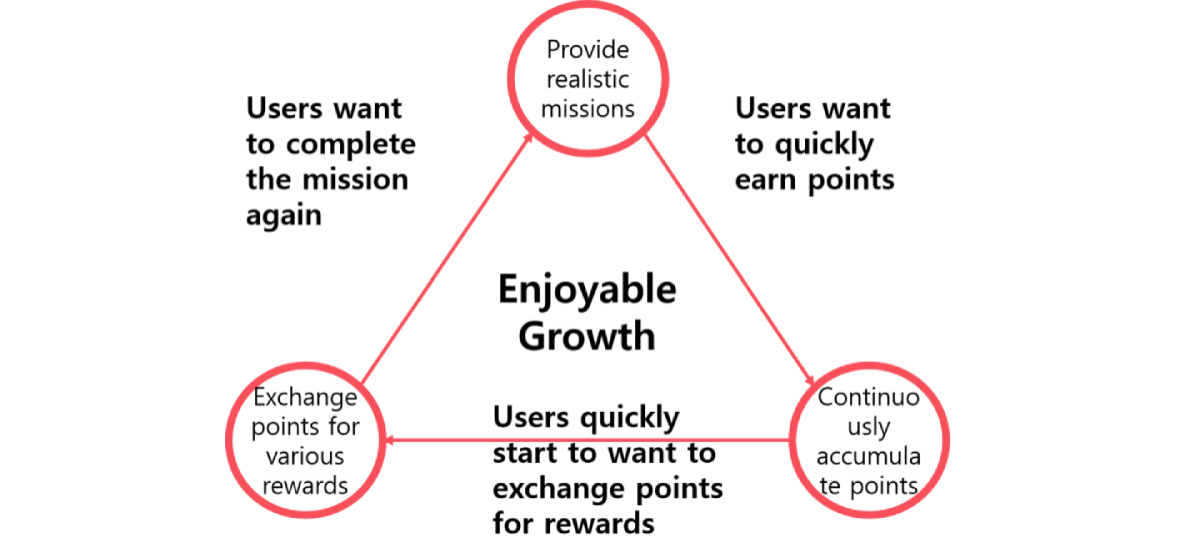
The role of points in gamified systems.

Points originated from a token economy that was used to induce changes in students’ behavior at home or in the classroom. Tokens were issued to encourage behavior that helped learning activities, and the students exchanged the collected tokens for rewards that were helpful for studying. The token economy positively affected learners’ self-reflection, improved their learning attitude, and stimulated participation in learning activities [16,17]. After 2011, when gamification was defined, the PBL system, which could add gameful experience to a token economy, was actively used, according to previous studies published between 2015 and 2020 [6,10,18]. Points provide learners with feedback by quantitatively showing feedback for learning activities. In a system described by Kim et al [14], learners received realistic missions from their instructors. The rewards that the learners received when they successfully performed these missions were points (Figure 1).

Points are accumulated when learners repeat a mission. The accumulated points represent the time and effort that the learners have invested in learning and show the role of stimulation on learners’ motivation. Through this process, learners set goals for learning, compete with themselves and others in good faith, pursue self-achievement, and behave as expected by the instructor, thereby allowing behavior to be corrected through affordance [10].

#### Problems With Points in Learning Environments

If the point system is applied without considering cautionary factors, it may negatively affect learning. The use of points promotes learning activities, but the unconsidered use of points without an appropriate purpose has the effect of nullifying the gameful experience that gamification provides to users. We identified 3 problems with points reported in previous studies.

The first problem is the accumulation of points over time. In the token economy, tokens could be exchanged for rewards. Various methods were considered to prevent inner motivation from weakening due to this use of rewards. In a gamified environment, however, problems occur when points are used related to how the points that are obtained and accumulated by the learners are shown on the leaderboard. According to Kim et al [11], learners experience a sense of anticipation and achievement when they earn points. However, as points are accumulated, the sense of anticipation and achievement weakens, and the meaning of the points gradually fades. Consequently, learners no longer engage in point-earning activities. Furthermore, even if rewards that can be earned with points are provided, they can also have an adverse effect on learners, because new rewards that can stimulate more powerful external motivation must be continuously provided [11].

The second problem with points is that they can emphasize differences between people. When adopting the PBL system, many instructors display points that the learners earn on a leaderboard. In the system described by Park and Kim [6], the points display on the leaderboard showed the total points that were rewarded when a learner successfully completed a mission or quest set by the instructor. The learners checked and compared the displayed points, stimulating a competitive spirit. However, if the learners were exposed to this competitive environment for a long time, it resulted in academic stress, and the system turned into a simple competition that negatively affected learners [19]. Thus, points lose their feedback function, and learners work against the purpose of the gamification set by the instructor [20]. This is because the feedback provided by the points recorded on the leaderboard is the sum of all learning activities, not the individual activities of a learner. Learners grow through step-by-step feedback. Therefore, feedback that cannot provide specific details does not constitute effective feedback for learners [6].

The third problem is the problem of equally distributing rewards. In general, learning activities are divided into individual and team levels. Instructors set team-level quests to develop soft skills, such as collaboration, cooperation, and communication. A quest is established in such a way that all team members complete it together. If the team members perform the quest in cooperation, they earn points as rewards. Here, the team members qualitatively evaluate the activities of the participants in the team activity even though they cannot quantitatively evaluate them. If all team members are equally involved in the team activities, then each team member does not object to receiving the same number of points. However, if some of the team members do not participate diligently, then the other team members will feel that there is an equity problem. Based on this, the team members determine the fairness of the reward provided to each member. If the same reward is awarded to the “free riders,” [21] who do not participate in the team activity and directly or indirectly impede the activities of other members, the other members perceive that the rewards are not fair and participate unenthusiastically in the learning activities. Further, if the rewards are the same for those who participate actively in the team activity but invest less time and effort compared to other learners because of personal ability issues, this also results in making them look like free riders to other learners.

## Methods

### Overview

In this section, we describe the development of a point-design framework to prevent the problems caused by points when instructors consider using gamification with the PBL system. The reason for developing such a framework is to ensure ease of use. A framework is a method of defining problems in engineering and logically explaining how to solve them [22]. The biggest advantage of a framework is that it reduces the gap between the field and academic perspectives. Therefore, we developed a framework that could address the problems of points and be applied in the field [15].

We designed a points application framework for gameful experiences in learning environments using 3 steps. The first step involved establishing 3 criteria to solve the problem of applying points in a learning environment, previously discussed in the literature review section.

The second step involved designing point-type criteria based on the criteria derived in the first step. The first step only derived a conceptual definition to solve the problem of points; however, it is also necessary to understand the users.

The final step involved defining the point types based on the results derived in the first and second steps. The point types were designed to be easily used by the instructor developing the gamified learning environment. To aid the understanding and use of the point types, we suggest examples of how to use the derived point types.

**Figure 2:**
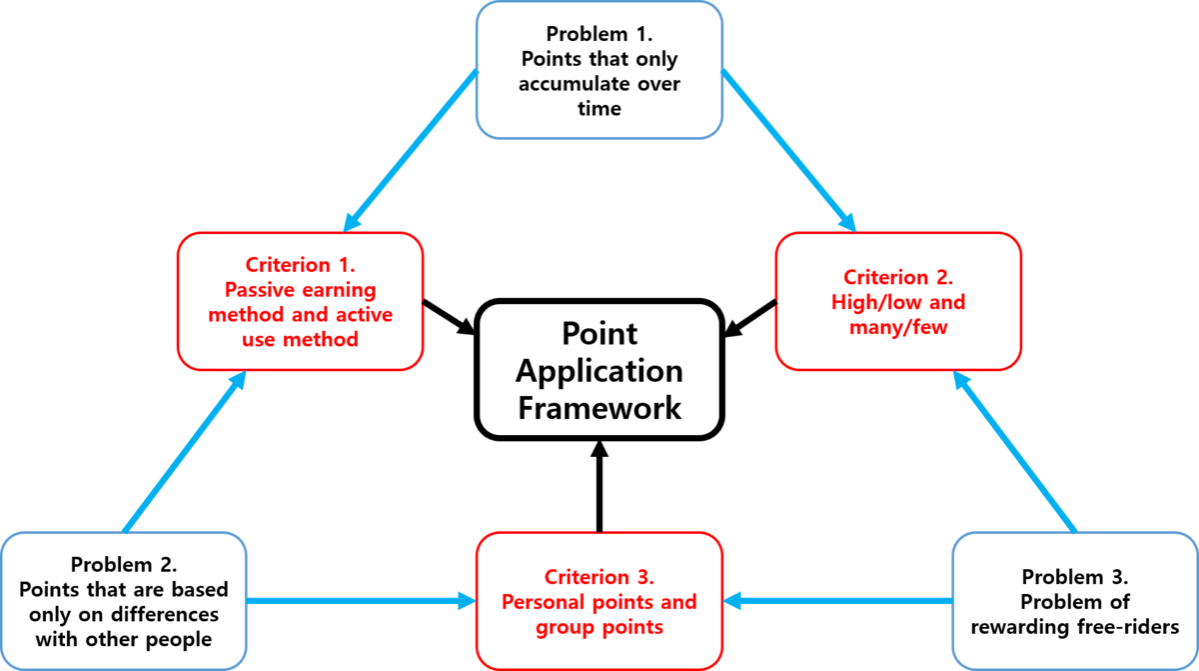
Point application framework.

Our point design framework intends to solve the problems mentioned in the previous section. We derived 3 criteria for the application of points. As shown in Figure 2, the framework is focused on a design to solve 3 problems identified by previous studies.

### Criterion 1: Passive Earning Method and Active Use Method of Points

The instructor sets a mission or quest for the learner. Points are awarded differently depending on the difficulty of the mission or quest that the learner must perform. Points are meant to recognize the time and effort that the learner invested in completing the mission or quest. From the learner’s perspective, they are an indicator of accomplishment, showing that the learner has completed a difficult job. The instructor should assign missions or quests to the learner repeatedly, so that the learner can master the knowledge. A problem arises in this process. The points earned by repeatedly completing missions or quests accumulate without being used. In the early stage of learning, the learner enjoys this, because the experience of earning points is new. However, with time, the joy derived from earning points diminishes.

The reason this phenomenon occurs is that points act as a reinforcement [12]. Meanwhile, the failure to provide better and better rewards leads to a degeneration of inner motivation [14]. From the standpoint of educators, it is practically impossible to periodically prepare better rewards. Reinforcements increase the probability that learners will perform the actions set by the educator just up to the point where the reward is given. Therefore, values other than reinforcement alone should be assigned to points.

The characteristics of points should be partially adjusted to use them as more than just reinforcements. The experience provided by points in the early learning stage is novel and stimulates a spirit of challenge. Therefore, the learners try to earn points. However, with time, they obtain points based on a sense of obligation because they are drawn to the familiarity of the points. Here, the experience, learning motivation, and sense of accomplishment are linked to familiarity [23]. The learners earn points because of this familiarity, and because the points become an “indicator” that displays their status on the leaderboard to other students, they no longer reflect the learners’ willingness to learn, and the learners become passive.

Points should also change the learners’ passive attitude to an active one. Conventional, passively earned points do not reflect learners’ willingness. If the learners can use the points according to their own will, they will have a different experience. For example, let us assume that a learner has completed many missions and quests and collected a total of 30 points. The instructor can allow the learner to exchange the points for information that is helpful for learning, as a reward. In this case, the learner used the reward based on their own will, and this was an activity that was performed according to the rule agreed upon by everyone who participated in the learning activity.

By using points based on their own will, learners can improve the learning experience. In this way, learners will realize that there is a relationship between their existence and that of other people, and this will motivate the learners to actively participate in learning [24]. Furthermore, the active use of points stimulates the cohesion of the structural relationship of learners while interacting with the educational environment set up by the instructor. This promotes learning activities, and as a result, points act as a genuine reinforcement [14,24].

In the end, the passive earning method of points should be utilized for conventional feedback functions and triggering competition in good faith, while the active use method should be used to improve the learning experience of learners and solve the problems caused by accumulated points to let the learners become immersed in studying. When learners successfully complete missions or quests using both the passive earning method and the active use methods, their self-efficacy will be maximized. Learners will believe that the next mission or quest can be successfully completed, and they will also believe in the value of the reward received after completing a mission or quest in the group that they belong to [14]. Through this process, the learners’ learning experience will improve, positively affecting their learning.

### Criterion 2: Significance of Numbers in Points—High/Low and Many/Few

Points have the characteristic of being numbers. Therefore, when they are displayed on the leaderboard, or when users interpret their meaning, they reflect the characteristics of numbers. Numbers can express both quality, as simple numbers, and quantity, as a number of tokens, coupons, or coins. Qualitatively, if player A has a higher score than player B, then we acknowledge that player A is the one who did better. Furthermore, players with a lower score will be motivated in their activities and will set the goal of breaking the record of the player with the highest score [25]. These are points in the “high/low” sense. Meanwhile, points can also be used quantitatively, as in the expression that player A has earned “many points” while player B has earned “few points.” Quantitative points are similar to tokens in the token economy system. In this system, if the intended activity of the educator is performed, tokens are received, and the earned tokens are used and exchanged for a reward [26]. For high/low points, scores can are displayed on a leaderboard, and users compare their scores to others’. Quantitative points allow a relative comparison of many/few points without a leaderboard, based on the quantity of points that a user possesses.

The high/low points displayed on the leaderboard provide intuitive feedback to the learner. Meanwhile, many/few points are related to operant conditioning. In a gamified learning environment, rewards are awarded differently depending on the activity that learners select. Here, points are used as a reinforcement to encourage learners to perform the activities intended by the educator. As time passes, the learners accumulate experience with points. The learners can use points for optional rewards by operant conditioning rather than as reinforcements. Based on operant conditioning, quantitative points encourage learners to actively participate in learning activities [27].

The concept of quantitative points is different from that of virtual currencies. A virtual currency is a type of game mechanic associated with the economic system of a game, and the educator must maintain fairness through currency balance [14]. It is also a kind of game mechanic used for compensation. However, quantitative points induce the action of exchanging points for rewards set by the educator using the concept of exchange. Therefore, with virtual currency, there is no price or benefit, and the exchanged rewards do not lead to the collapse of the learning balance between learners. Therefore, quantitative points and virtual currencies are different concepts.

### Criterion 3: Personal and Group Points

Slavin [28] insists that systematically establishing a reward structure for learners is important because, according to their analysis, if an environment for sharing the learning activity process and results with other learners is established, a personal-level reward structure will increase learners’ performance. Considering the social aspect of learners, however, a cooperative reward structure is needed. This is because learners are attracted to the soft skills required for the interaction between learners, including skills such as learning and building social rapport, rather than simply acquiring knowledge. Ultimately, the classroom atmosphere can be changed. Social activities among learners have positive effects on communication, listening ability, problem-solving skills, and learning motivation [29]. Therefore, educators should simultaneously consider both the personal level and the group level when they design learning activities.

No major problems occur with the personal points received after completing personal-level learning activities, because the learner is rewarded in proportion to the time and effort invested. However, the fairness of rewards is important in activities where groups of 2 or more people participate. Suppose student A performed a group activity as well as they could, but student B did not. If they both receive the same level of reward, student A will not participate properly in subsequent learning activities. In this process, student A loses the satisfaction and value of the reward, because their belief and expectation in the the fairness of the reward are damaged, and its significance is lost. Due to this damage to motivation, the possibility of lukewarm participation will increase [30]. Therefore, learning activities should be subdivided, and personal points and group points should be configured to determine the points awarded to the group and the points awarded to individuals, even for group activities.

### Principle of Solving Problems in the Point Design Framework

The 3 criteria derived in this study solve the 3 problems based on the following principle: The passive point-earning method, in which points are only accumulated over time, is associated with the characteristic of numbers that they can be high or low, because the points are recorded on the leaderboard to deliver feedback. Based on how high or low the score is, the participation level in the learning activity can be understood, and the learning level of the learner can be understood. Meanwhile, if the active point-use method is added to the passive point-earning method, the problem arising from point accumulation disappears. By exchanging the points earned for a reward offered by the educator, learners perceive that they were given a new means or privilege to participate actively in learning activities. Therefore, if the passive earning method and the active use method are combined and applied to points, and the meaning of high/low and many/few points is used, a new experience can be delivered to learners. Furthermore, while classifying the size of the rewards at the personal and group levels, if additional missions or quests are set up so that personal-level rewards can also be received in a group learning activity and additional rewards are configured to be awarded differently depending on the participation level of individuals in the group activity, the free-rider problem can be partially solved. Additionally, the ability to make strategic choices can be granted to learners through various point types. When learners choose their own learning activities to earn points, they begin to understand the meaning of points. Here, if learners choose learning activities with point types related to the active use method, they will earn rewards that are helpful for the learning activity. For strengthening competence and competition in good faith, however, learners prefer learning activities in which points can be passively earned. As such, learners will strategically participate in learning, which is expected to improve their learning attitudes.

## Results

### Point Types and Examination of Actual Applied Cases

**Figure 3:**
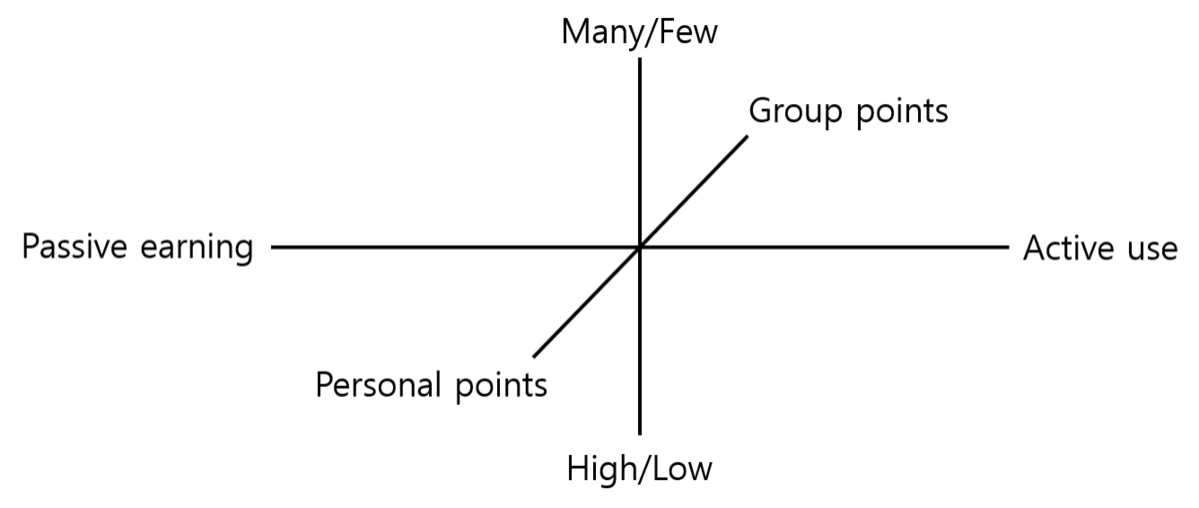
Point type criterion.

We derived a point application framework based on the 3 criteria. Based on the framework, we derived 8 types of points. The point types were classified and derived based on the criteria shown in Figure 3.

**Figure 4:**
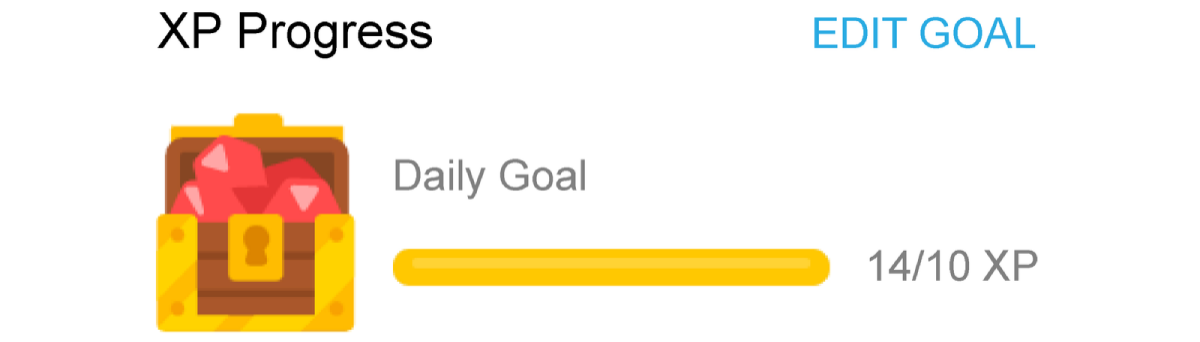
Experience point example (Duolingo).

The first point type is experience points (EXP). EXP represents the player’s activity numerically. It is a game mechanic related to the user’s level. It can be earned passively and compared as a many/few–type number. Furthermore, EXP is given as a personal reward. As players collect more EXP, their level goes up, and the time and effort that they invested can be estimated based on how high their level is. Figure 4 shows an example of EXP in Duolingo (Duolingo, Inc), a language-learning platform. This platform expresses experience points as “XP.” If the specified amount of learning is completed, XP can be earned. The user cannot control their XP. Learning motivation is stimulated by achieving a higher level through XP.

**Figure 5:**
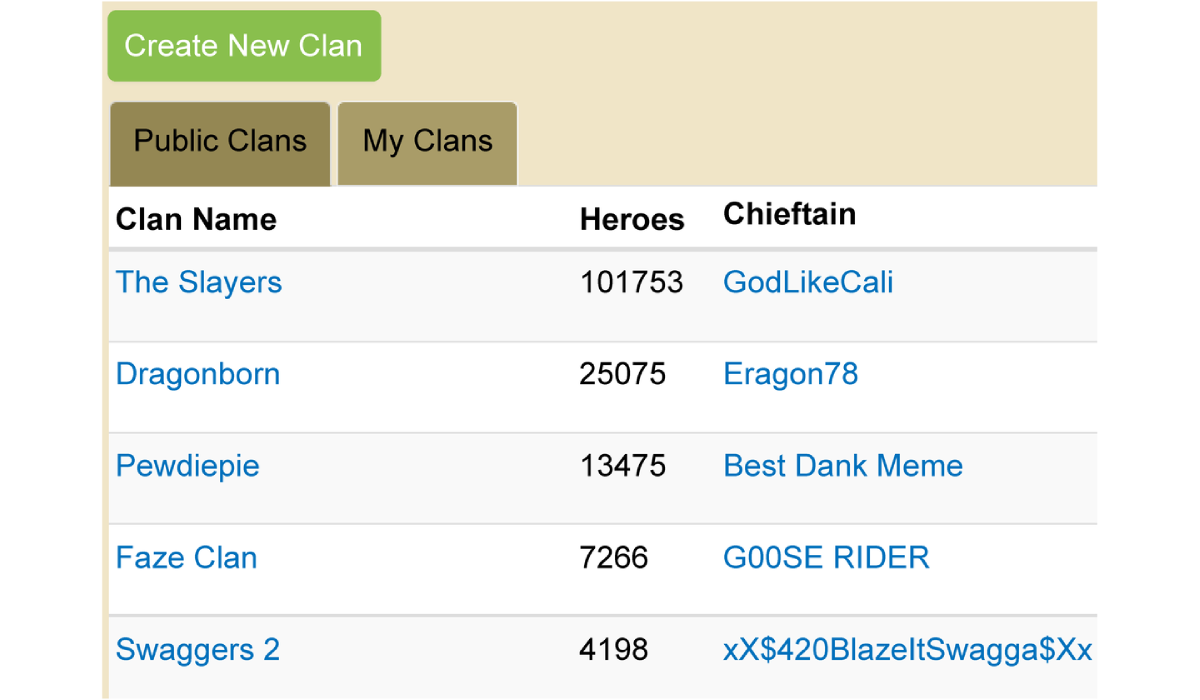
Guild experience point example (CodeCombat).

The second point type is guild EXP. Guild is a word that originates from the guild system that craftsmen established to train apprentices in medieval Europe. Learners belonging to a guild expand their knowledge and skills by interacting with guild members [30]. Guild EXP is given as a group reward, unlike regular EXP. Guild EXP is also used with levels and uses a structure in which guild members work hard to accumulate guild EXP to increase the guild’s level. As the guild’s level increases, value-added effects increase. For example, the guild becomes envied by other players, who set a goal to join the guild. Figure 5 shows a clan leaderboard of CodeCombat (CodeCombat Inc), a platform for programming-education content. This platform uses the word “clan” instead of “guild,” and the number of heroes corresponds to guild EXP. The number of heroes is derived from the missions and quests completed by learners belonging to the clan.

The third point type is ability points. Ability points can be earned passively, and the points can be compared as high/low–type points. Furthermore, they are personal reward points. Ability points visually show that knowledge or skill in the relevant area has increased as a result of actively performing learning activities when learning various subjects, such as English, mathematics, and leadership. For example, if student A studies a higher level of mathematics compared to other subjects, the instructor raises the logic level of student A to the next level. Based on this process, the learner’s competence can be represented numerically by changing the learner’s abilities (eg, wisdom, power, and logic) [31]; the abilities that the learner can obtain by studying are set to dexterity, intelligence, and discipline. The relevant ability points can be increased after completing actual missions and quests.

**Figure 6:**
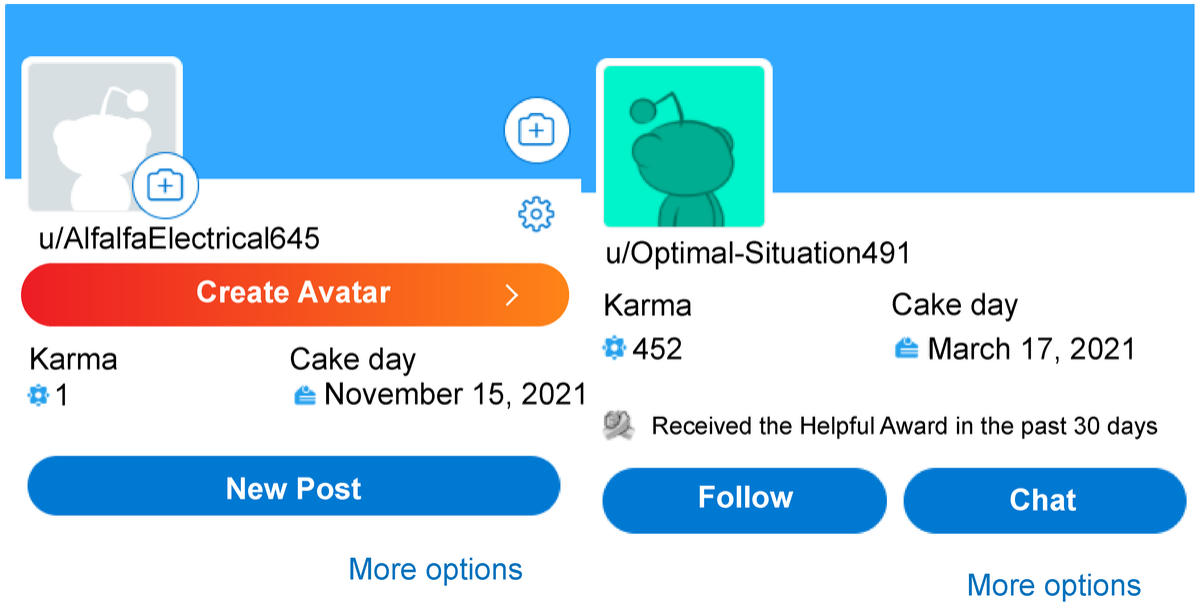
Karma point example on Reddit.

The fourth point type is karma points. Karma points can be earned passively and compared as high/low points, but they are group reward points. If some members in a team of 2 or more learners successfully complete a mission or quest, all members of the team receive points as a reward. Karma points can stimulate peer companionship, and using them as a feedback device among group members induces changes in the learning behavior of the beneficiary learners [32]. In the process, the learners will have a positive learning experience and feel fulfillment, pride, and satisfaction with the learning activity based on the bonds they form with other learners [14,33,34]. Figure 6 shows a user screen from Reddit (Reddit Inc), a social media service. When a user posts or shares something meaningful to other people, other users send karma points as a way of appreciating the user’s activity. When users’ karma points are high, it implicitly acknowledges that they have provided meaningful information to many people.

**Figure 7:**
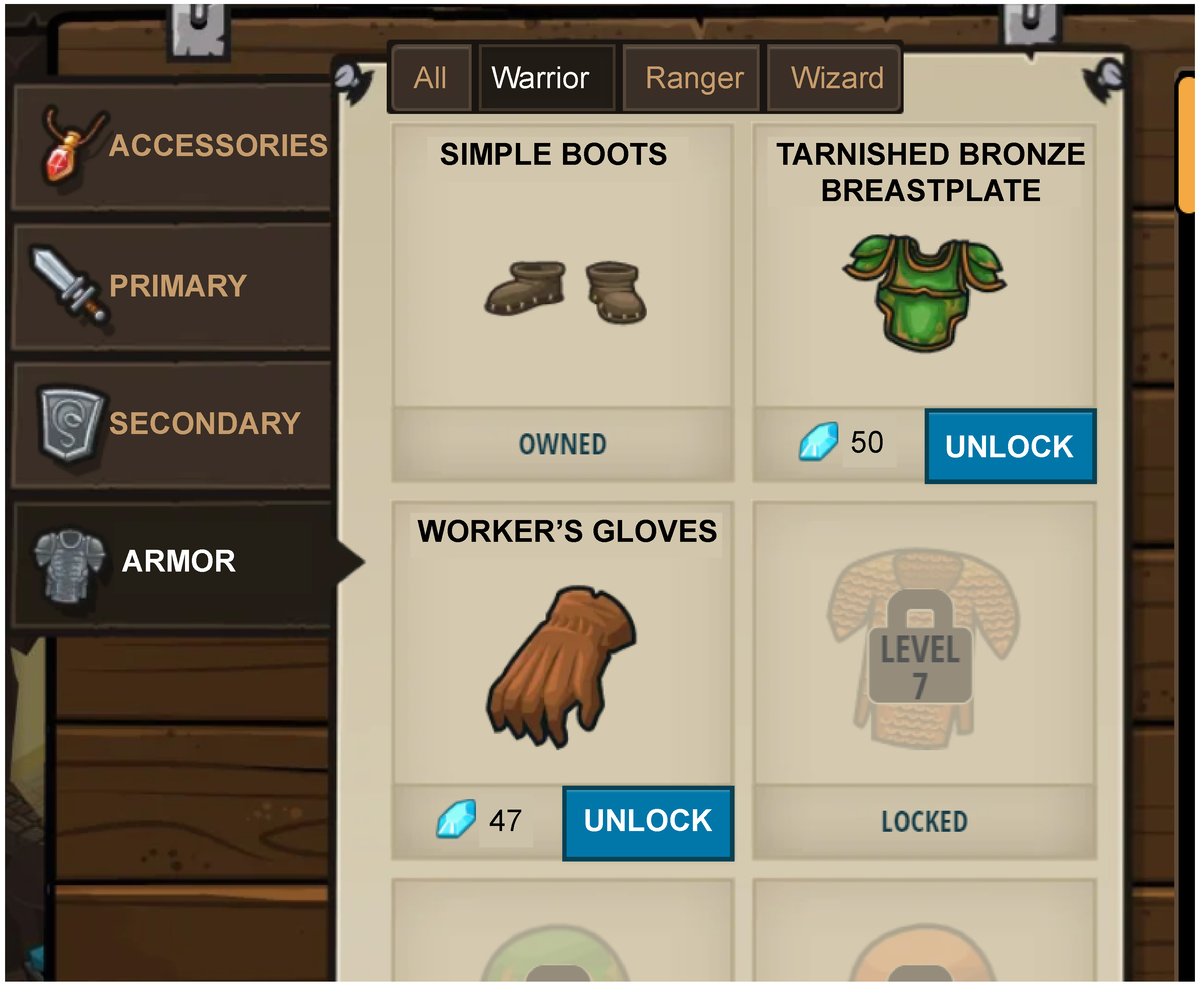
Exchange point example (CodeCombat).

The fifth point type is exchange points. Those who possess points can actively use them, and the more points, the better. They can also be exchanged for something for oneself, such as virtual items, benefits, or privileges that are helpful for learning according to the reward system set by the educator. Exchange points differ from virtual currencies; point users use them based on their own thought and will. Figure 7 shows a virtual shop in CodeCombat. Players can earn blue crystals as points by performing missions or quests and exchanging the earned points for items that can make their avatar more powerful.

The sixth point type is group exchange points. Unlike exchange points, the entity that uses the points is not the individual, but the group, based on their collective opinion. The privileges, benefits, and virtual items exchanged using points should be beneficial to all members. This point type is commonly used in massively multiplayer role-playing games, and the group leader engages in discussions with the members to exchange the points for items or effects they need.

**Figure 8:**
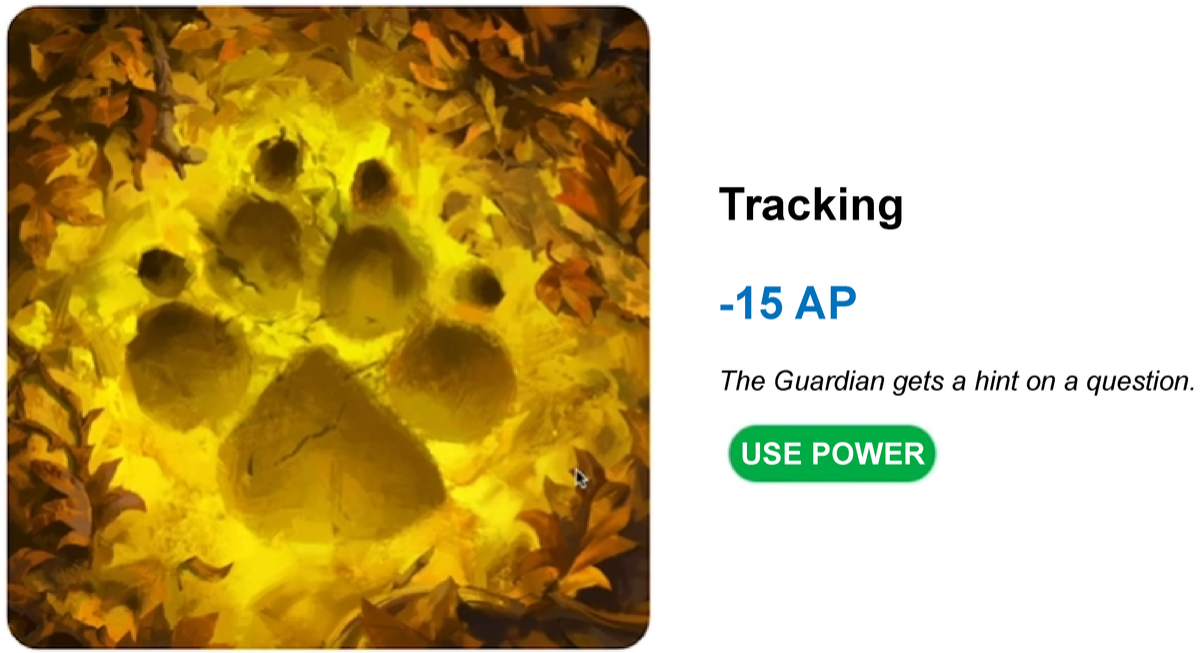
Skill point example (Classcraft).

The seventh point type is skill points. Skill points are used when encouraging learners to obtain certain knowledge or skills. For example, suppose the learners wish to learn theory A, and the instructor deliberately sets up relevant missions and quests. Here, the instructor classifies the theory A into levels 1, 2, and 3. If learners complete a mission or quest related to learning theory A several times, they can attempt a higher level of mission or quest. If the learner has successfully completed the level 1 mission or quest, the learning level of theory A is raised by 1 point. If all 3 points have been earned by repeating this process, other people acknowledge that the learner understands theory A to a corresponding degree. Furthermore, when the skill points increase, privileges or benefits may be granted to help the learner with learning. For example, a learner who has achieved 2 skill points for theory A may help another learner when performing the level 1 mission or quest or use the privileges or benefits acquired to overcome a difficulty that they face. Figure 8 shows an example of skill points in Classcraft (Classcraft Studios Inc), a class management program. Skills exist in the form of a tree, and a user’s points in a lower hierarchical skill must reach a certain level to use an upper hierarchical skill. If skill points increase, better benefits or privileges can be experienced.

The eighth point type is peer review points, which are used when learners in teams of 2 or more evaluate each other’s learning activity. Whereas karma points are emotionally linked to the members, peer review points are used when evaluating peers. The members of other teams cannot use them, and these points are used when evaluating the members of the same team based on the experience of interacting with them. Peer review points create bonds between members by letting them provide feedback to each other, and based on the bonds, learners build their own learning experiences. This process positively affects learning [35].

## Discussion

Points are a gameful experience–delivering element that can be used in the largest proportion of the educational environment. The performance of learners can be visually displayed through points, and the displayed points can be used as the basis for learners to recognize each other’s progress. Educators use rewards to set up goals and stimulate learning motivation so that the learners can study smoothly. The learners recognize the points set up by the instructor and participate in learning activities, whereby it is hoped that the learning attitude of the learners will improve. However, improper use of points can adversely affect the learners and the educator. For example, the improper use of points may cause learners to nullify gamification and participate in learning activities to earn points without a genuine purpose.

The aim of this study is to suggest guidelines, by developing a point design framework, for educators considering the application of points that will help them avoid mistakes. To this end, we explore the educational effects of points and the definition of the framework and have presented 3 problems arising due to points. To solve these 3 problems, we have proposed 3 design criteria and 8 types of points based on past studies and actual cases.

We recommend the following approaches to educators who are considering applying points and using the point application framework developed in this study. First, the reinforcement and reward characteristics of points must both be reflected. If points are used as simple reinforcements, the learners will only repeat the learning activity intended by the instructor. Consequently, there is a high possibility that the learners will not experience joy as time passes. For sustainable learning, therefore, the point application framework developed in this study should be used to design points that have both reinforcement and reward characteristics. Second, attention should be paid to pointsification. It is necessary to choose actions through which the learners earn points, but if the points have more value than the learning activity itself, the learners will try to earn points without a true purpose. Therefore, points should use a sophisticated design based on the point application framework. Otherwise, learners will feel lethargic and find problems regarding fairness, and if these problems are not resolved, the learners will give up on learning. Last, the point application framework should be used to prevent the overjustification effect. Even high inner motivation in a learner may become weakened if the learner is continuously exposed to external rewards. In this case, the learner may blame the rewards for their change in attitude [36]. The point application framework and mission or quest settings should be considered at the same time to prevent the overjustification effect. Points should not be used as simple rewards, and an environment that promotes the stimulation of learning motivation should be established based on operant conditioning [37].

There were limitations of this study that suggest future research directions. If a badge application framework and a leaderboard design framework are used, it is possible to develop a PBL system that promises educational effects while effectively delivering a gameful experience. However, there is a lack of studies on missions and quests related to PBL. Research on methods for setting up missions and quests for the effective education of learners is insufficient, and this aspect is not covered in this study. Further research is required to develop a methodology to set missions and quests or a framework that will facilitate the balanced education of learners.

## Abbreviations

EXP: experience points
MZ: millennials and Generation Z
PBL: point, badge, leaderboard
